# Vocal tract analysis in patients with vocal fold nodules, clefts and cysts

**DOI:** 10.1016/S1808-8694(15)30777-1

**Published:** 2015-10-19

**Authors:** Raquel Buzelin Nunes, Andrea Moreira Veiga de Souza, Andre de Campos Duprat, Marta Assumpção de Andrade e Silva, Rejane Cardoso Costa, Juliana Gomes Paulino

**Affiliations:** 1 Master's degree in speech therapy, Pontifícia Universidade Católica de São Paulo. Speech therapist; 2 Otorhinolaryngologist at the Espaço da Voz, Belo Horizonte. Otorhinolaryngologist of the Prefeitura de B.H; 3 Professor of the Faculdade de Ciências Médicas, São Paulo. Santa Casa de São Paulo. Otorhinolaryngologist; 4 Professor at PUC, São Paulo. Speech therapist; 5 Otorhinolaryngologist, Audiomigmg; 6 Otorhinolaryngologist, Instituto de Otorrinolaringologia de Minas Gerais. Pontifícia Universidade Católica de São Paulo

**Keywords:** dysphonia, endoscopy, vocal fold

## Abstract

The supraglottic plan represents an important dimension in vocal production, and its characterization is very important in the evaluation and treatment approach of dysphonic individuals.

**Aim:**

to check if certain glottic configurations are related to specific adjustments in the vocal tract. To use nasal and laryngeal fibroscopy to assess the frequency of supraglottic vocal tract adjustments in dysphonic women with nodules, clefts and cysts.

**Methods:**

We assessed 31 dysphonic women, with age ranging between 18 and 45 years, with vocal alteration and a diagnosis of nodules, middle-posterior cleft and cyst, and we carried out a summarized evaluation of the sensory-motor and oral systems and the patients were submitted to video-laryngostroboscopy and nasal and laryngeal fibroscopy. Three distinct groups were selected: patients with bilateral nodules, clefts and cysts, with similar glottic configuration. Their vocal tracts were visually analyzed through exams of nasal and laryngeal fibroscopy, by speech and hearing therapists and otorhinolaryngologists, checking the following parameters: supraglottic constriction, larynx vertical mobility, pharyngeal constriction and tongue mobility. The data was statistically described and treated.

**Results:**

during visual analysis we did not find statistically significant differences which would separate the glottic alterations groups.

**Conclusion:**

There was no correlation between supraglottic tract adjustments with any particular type of glottic alteration. These are individual behaviors that generate adjustments and justify the different vocal qualities in patients with the same type of laryngeal alteration.

## INTRODUCTION

Voice is an important communication mode among human beings. The glottis, supraglottic area and the respiratory system are essential for producing voice.

The source-filter model for vowel production emphasizes the glottic and supraglottic dimensions that are responsible for phonation (Fant, 1970). According to this author, the source is glottic action, or sound generated by the vibration of vocal folds, and the supraglottic area operates as a filter by means of resonance. Depending on the configuration of the supraglottis, which is directly related with the opening of the mouth, the position of the tongue, pharyngeal constriction, and the palate, different harmonic groups are amplified; this generates the formants and voice quality.

The supraglottic plane is an important aspect of voice; it should be characterized when assessing and treating dysphonic individuals, since these patients use compensatory strategies when producing voice to overcome anatomic and/of functional alterations.^2^ Medical and phonoaudiological research, however, has focused mainly on laryngeal images, and generally have not taken in to account the supraglottic plane.

Many papers have been written on the supraglottic plane in normal individuals,^3^, ^4^, ^5^, ^6^, ^7^, ^8^, ^9^, ^10^, ^11^^,^^13^^,^^14^ but few have studied dysphonic subjects.^2^^,^^15^, ^16^, ^17^, ^18^

Patients with specific vocal fold lesions often benefit from the possibility that vocal tract plasticity may change the acoustic features of voice. This is also evident when the voice of patients improves markedly following speech therapy, although images of the glottis are unchanged.

Because similar glottic configurations in dysphonic patients may yield different qualities of voice, it was thought that possibly the supraglottic tract may be responsible for such differences in voice. The question, then, was “How could similar glottic images generate voices that were so different?”

With this in mind, there was an interest in studying the behavior of the vocal tract in dysphonic female patients diagnosed with similar vocal fold nodules, cysts and clefts.

## OBJECTIVE

The purpose of this study was to verify the frequency and to compare the adjustments made by the supraglottic tract in three groups of female dysphonic patients diagnosed with nodules, clefts and cysts, and with a similar glottic configuration, using nasofibrolaryngoscopy.

## METHOD

The Research Ethics Committee of the Pontificia Universidade Catolica de São Paulo approved this study (no 0164/2003).

From an initial sample of 40 subjects, 31 female subjects aged from 20 to 45 years were selected; this age range is considered as that with the highest voice efficiency. The subjects were seen at a private otorhinolaryngology clinic in October and November 2004, and complained of voice changes; all had a videolaryngostroboscopic diagnosis of nodules, middle-posterior clefts or cysts.

In this study the nomenclature for laryngeal alterations defining nodules as generally bilateral benign lesions occurring on the anterior portion of the vocal folds was applied. Middle-posterior clefts were geometric images of the remaining space in the rima glottidis during phonation, when the opposite vertex to the base generally meets the middle third of the vocal folds. Cysts were localized closed cavities within vocal folds.^19^

Inclusion criteria: female patients complaining of altered voice for over three months, not undertaking speech therapy, and never having undergone laryngeal microsurgery or any prior voice therapy, to avoid influencing the assessment in general.

Exclusion criteria: subjects with mental disorders or an altered oral sensory-motor system that could interfere with the assessment, such as: severely altered lip, cheek or tongue tone or mobility, class two or three occlusion disorders, dental arcade faults, upper or lower airway alterations on the day of the examination, use of fixed appliances or dental prostheses (according to Oliveira and Pinho, 2001,^20^ these interfere significantly with speech adjustments). The researcher thus elaborated an identification protocol for subjects and the sample selection consisting of data on age, complaint, habits, water intake, use of voice, profession, and a summarized evaluation of the oral sensory-motor system: lips, tongue and cheek (tone and mobility), altered dental occlusion, use of dental prostheses or orthodontic appliances, joint (ample, closed, locked, “smile”, vertical), and face (long, short, medium, symmetrical or asymmetrical).

Subjects were informed about and signed a free informed consent form.

An otorhinolaryngologist carried out direct laryngoscopy with a Mashida 30° or Explorent 70° rigid telescope coupled to a Toshiba micro-camera, a Bruel & Kjaer stroboview source of stroboscopic light; the examination was recorded on a JVC super VHS videocassette with an LG 20” television monitor for diagnosing the lesions. The physician introduced the laryngoscope into the patient's mouth and asked the patient to issue the prolonged vowel /ê/, /i/ and to carry out inspiratory phonation. Local anesthesia (10% neocaine) was applied when patient's reported discomfort.

The next step was nasofibrolaryngoscopy using a Mashida ENT 30 P III fibroscope coupled to a Toshiba micro-camera and an Alphatron light source; the device was introduced into the nostril and placed immediately below the soft palate to check vocal tract adjustments. Patients were instructed to issue the prolonged vowel /a/ and to speak spontaneously. Examinations were recorded on VHS tape using a JVC super VHS videocassette recorder.

Examinations (direct laryngoscopy) were shown separately at a later moment to two other otorhinolaryngologists. The reports were compared, and those with the same diagnosis were kept in the sample. Of 40 examinations with coincident reports, otorhinolaryngologists selected those with similar glottic findings: the same types of clefts of proportional sizes, the same types of lesions with the same types of associated clefts, similar size and shape and similar location on vocal folds. As a result of this process, nine examinations were excluded; thus, 31 examinations were left, which were allocated to three groups according to the similarities. The first group consisted of examinations with bilateral nodules (group 1); the second group consisted of examinations with cysts (group 2); and the third group consisted of examinations with clefts (group 3). The similarity of examinations is of extreme importance in this study; the choices, thus, was done using this method, rather than clinical incidence.

Three otorhinolaryngologists and three speech therapists assessed the nasofibrolaryngoscopy examinations in each of the abovementioned three groups. These professionals had more than five years of experience each in the study of voice, to provide reliability in judgment.

A protocol adapted to this study was applied to investigate the following conditions: presence or absence of supraglottic constriction; vertical motility of the larynx; presence or absence of pharyngeal constriction; and position of the tongue.

The results of the visual analysis were analyzed descriptively, followed by a statistical analysis consisting of a comparison with the Kruskal-Wallis test to verify the differences among groups. The significance level was 5% (p= 0.050).

## RESULTS

There were 31 female patients allocated to three groups, as follows: 10 patients diagnosed with nodules, 10 patients diagnosed with clefts, and 11 patients diagnosed with cysts. Table 1 shows the result of the visual analysis in the three groups.

**Table 1:** Description of the visual analysis (nasofibrolaryngoscopy) of the frequency of supraglottic vocal tract adjustments in three groups (nodules, clefts, cysts).

	Nodules		clefts		cysts	
Supraglottic constriction:	N	%	N	%	N	%
No constriction	3	30	4	40	3	27,3
Middle constriction	0	0	1	10	2	18,2
Antero-posterior constriction	6	60	5	50	4	36,3
Antero-posterior and middle constriction	1	10	0	0	2	18,2
Vertical laryngeal motility:						
ample	2	20	1	10	0	0
medium	3	30	6	60	3	27,3
restricted	5	50	3	30	8	72,7
Pharynx:						
no constriction	4	40	6	60	5	45,5
amplified	0	0	0	0	0	0
circular constriction	3	30	2	20	4	36,3
lateral constriction	3	30	2	20	2	18,2
Tongue:						
anterior	1	10	3	30	0	0
neutral	9	90	7	70	11	100
total	10	100	10	100	11	100

A comparative analysis was done by applying the Kruskal-Wallis statistical test for the adjustments found in each group; there was no statistically significant difference among the groups. The adjustments that were observed in subjects while issuing a sustained vowel and connected speech (Table 2).

**Table 2:** Description of the comparison among groups of adjustments when issuing a sustained vowel and connected speech, followed by the significance level (p < 0.050)

ADJUSTMENT	Significance (p)
No supraglottic constriction	0.815
Middle supraglottic constriction	0.619
Antero-posterior supraglottic constriction	0.644
Ample laryngeal motility	0.313
Medium laryngeal motility	0.573
Restricted laryngeal motility	0.229
Pharynx with no constriction	0.660
Amplified pharynx	1.000
Circular pharyngeal constriction	0.988
Lateral pharyngeal constriction	0.518
Ample tongue	0.125
Neutral tongue	0.125
Tongue with elevated dorsum	1.000

Kruskal-Wallis test

## DISCUSSION

A daily clinical observation of dysphonic patients with similar diagnoses and different qualities of voice generated an interest in verifying whether the status of the supraglottic plane could explain such differences. Subjects work in different professions and have unique individual behaviors and anatomic features. The first challenge, therefore, was to select subjects with similar glottic features. This difficulty meant that there were only 31 subjects in the study sample, which were allocated to three groups according to the glottic configuration. These groups were defined based on the aim of investigating whether subjects had similar or dissimilar behaviors of the supraglottic plane.

The sample consisted only of females because of the high rate of dysphonia in women. Differences in glottic proportions, and the length and shape of the supraglottic tract were the reasons why both sexes were not chosen.

Three otorhinolaryngologists and three speech therapists with expertise in voice carried out the visual analysis of the supraglottic tract. A consensual assessment was chosen to increase the reliability of the results. The referees found it difficult to assess the type of pharyngeal constriction and especially the position of the tongue during the visual analysis. A joint evaluation was very relevant, underlining the importance of multidisciplinary work in which knowledge exchanges and reflection leads to novel approaches and professional development in both areas of expertise.

The age in the sample ranged from 20 to 45 years, which is similar to that found in other studies.^3^, ^4^, ^5^, ^6^^,^^15^^,^^16^^,^^21^

Other papers have also included only female subjects, probably because dysphonia is more prevalent in this sex.^4^^,^^6^^,^^15^^,^^16^^,^^21^

Campiotto,4 Campos,5 and Titze et al.^8^ studied professionals that depend on their voice. In our study, ^23^ of 31 subjects were professionals that depended on their voice; 11 were teachers and the others were secretaries, salespersons, a lawyer, a telemarketing operator, singers and actresses. It is thought that supraglottic tract adjustments occur more often in such professionals, since benefits may accrue at times from the use of voice. It is common to see teachers using pharyngeal constriction to help project their voices. Singers usually undertake adjustments of pharyngeal constriction, antero-posterior constriction and ample laryngeal vertical motility to increase the volume of their voices, to project their voices, or to sing a specific type of music; and actors may move their tongues in many ways to yield different types of voices. These efforts are generally not seen in subjects that do not use their voices professionally for speaking or singing.

The following parameters were noted in the visual analysis of the supraglottic vocal tract: supraglottic constriction, laryngeal motility, pharyngeal constriction, and position of the tongue. Studies that also assessed these points were those of Campiotto^4^ (of singers), Campos^5^ and Bezerra^6^ (of actors), and Denunci^22^ (of children, comparing voice quality before and after adenoidectomy and tonsillectomy.

The visual analysis revealed a marked variety in adjustment frequency in the three groups (Table 1). Adjustments of the tract were not specific for a given glottic configuration; thus, the features of the supraglottic tract could not be correlated with each type of glottic change.

An attempt was made in this study to observe whether specific glottic configurations correlated with specific adjustments of the supraglottic tract. However, the comparison of adjustments found in the three groups revealed no statistically significant differences among them (Table 2). Individual behaviors generate the adjustments that explain different voice qualities in patients with the same type of laryngeal condition. Hara and Veloso's^15^ study suggests this possibility.

It should be noted that during the visual analysis of supraglottic adjustments, the researchers realized the importance of carrying out nasofibrolaryngoscopy in the diagnosis and treatment of dysphonic subjects, as well as videolaryngostroboscopy. Unfortunately, an assessment of supraglottic structures is rarely done in medical routine; however, a diagnosis of supraglottic adjustments is important in the care of dysphonic subjects, especially those that use their voice professionally.

This study was an attempt to focus on the supraglottic tract, rather than looking at glottic alterations, given that the tract is usually left aside. Voice is the product of a connection between a source (glottis) and a filter (vocal tract); voice quality, thus, should not be correlated only with glottic changes.

This study revealed a deficiency in the use of available methods and a need to create new protocols to evaluate all aspects of voice.

## CONCLUSION

A visual analysis of the supraglottic tract in dysphonic women with different glottic configurations revealed that:

Adjustments of the tract are not specific for a certain glottic configuration, and the features of the supraglottic tract may not be correlated with each type of glottic alteration.

No statistically significant difference was found in a comparison of adjustments found in the three groups. Individual behaviors generate such adjustments, which explains the different qualities of voice in patients with the same type of laryngeal condition; therapy should, thus, be individualized.
